# Acute effects of three pulmonary reexpansion modalities on thoracoabdominal motion of healthy subjects: Randomized crossover study

**DOI:** 10.1371/journal.pone.0213773

**Published:** 2019-03-19

**Authors:** Rêncio Bento Florêncio, Andrea Aliverti, Marina Lyra Lima Cabral Fagundes, Ilsa Priscila dos Santos Batista, Antônio José Sarmento da Nóbrega, Vanessa Regiane Resqueti, Guilherme Augusto de Freitas Fregonezi

**Affiliations:** 1 PneumoCardioVascular Laboratory, Hospital Universitário Onofre Lopes, Empresa Brasileira de Serviços Hospitalares (EBSERH), Departamento de Fisioterapia, Universidade Federal do Rio Grande do Norte, Natal, Brazil; 2 Laboratório de Inovação Tecnológica em Reabilitação, Departamento de Fisioterapia, Universidade Federal do Rio Grande do Norte, Natal, Rio Grande do Norte, Brazil; 3 Dipartimento di Elettronica, Informazione e Bioingegneria, Politecnico di Milano, Milan, Italy; University of Notre Dame Australia, AUSTRALIA

## Abstract

**Background:**

Chest physiotherapy can be an alternative to increase lung volumes through pulmonary expansion therapies, but there is still inconsistency in the literature in order to determine which device can promote a greater volume increase at the expense of a better ventilatory pattern. Therefore, the aim of this study was to evaluate and compare the chest wall kinematics of healthy subjects submitted to the use of three different devices for pulmonary reexpansion.

**Methods:**

Chest wall compartmental and operational volumes, breathing pattern and thoracoabdominal asynchrony were evaluated in 12 healthy individuals through optoelectronic plethysmography during quiet breathing, pulmonary reexpansion and recovery. Three different devices (volume-oriented incentive spirometer–IS-v; positive expiratory pressure–PEP; and incentive spirometer volume and pressure oriented–IS-vp) were administered in a random order with at least 48h between the devices.

**Results:**

A greater volume variation in the chest wall and its compartments was observed when the IS-vp was used in comparison with the other devices (p<0.05). Furthermore, the IS-vp mobilizes a greater amount of volume accompanied by greater synchronism between the compartments when compared to IS-v (p <0.05).

**Conclusion:**

The IS-vp may be able to increase total and compartmental chest wall volumes, as well as improve synchrony among compartments when compared to IS-v and PEP devices, thus constituting an important tool for treating patients with restrictive ventilatory pattern.

## Introduction

Several situations in clinical practice favor subjects developing reduced lung volumes, characterizing a restrictive respiratory pattern [1]. Current evidence has already described that several patterns can be observed in the postoperative period of cardiac and abdominal surgeries [2,3], in patients with cystic fibrosis [4], obesity [5], Parkinson’s disease [6] and post-stroke [7]. The reduction in pulmonary volumes can promote pulmonary complications in the postoperative period and leads to worse quality of life for individuals, since simple tasks in daily life become impaired by lower ventilatory efficiency [8].

In this context, chest physiotherapy appears as a tool to increase the lung volumes of subjects who present a restrictive thorax, using pulmonary reexpansion techniques [9]. Several techniques and devices are recognized in recruiting the collapsed alveolar areas due to the restrictive pattern. Among them, the literature highlights those that promote this recruitment at the expense of an increase in the transpulmonary pressure gradient due to reduced pleural pressure, such as breathing exercises and incentive spirometer (IS); and those that increase alveolar pressure, with an emphasis on positive expiratory pressure (PEP) [10].

Recently, a group of researchers demonstrated that the combined use of IS with PEP in patients undergoing myocardial revascularization was shown to be effective in improving dyspnoea and quality of life in the subjects [11]. However, the literature still lacks evidence to demonstrate which technique is superior when used alone, as well as whether the combination of techniques is effective in increasing volume variations of the CW evaluated by optoelectronic plethysmography (OEP). It is important to emphasize that the OEP is a device with good accuracy that provides valid and reliable measures of CW volumes and its compartments, as previously described [12]. The present study is also justified by the physiological importance of the response to increased pulmonary expandability in the prevention and treatment of several lung diseases that evolve with a restrictive respiratory pattern. In view of the above, the objective of the present study was to evaluate and compare the volume variations of CW and thoracoabdominal motion from the analysis of the synchrony among chest wall compartments of healthy subjects submitted to using three different devices for pulmonary reexpansion.

## Methods

### Sample

This crossover study was conducted in the PneumoCardioVascular LAB (HUOL-EBSERH/UFRN) according to Consolidated Standards of Reporting Trials (CONSORT) guidelines [13] and approved by the Ethics and Research Committee of HUOL-EBSERH, under the protocol 2.666.702. All subjects gave written informed consent in accordance with the Declaration of Helsinki.

A convenience sample of healthy subjects of both genders aged 18 to 30 years with no history of cardiopulmonary or neurological diseases and/or comorbidities, no history of smoking, and who presented a body mass index (BMI) value within the normal range (18.5 ≤ BMI ≤ 24.99 kg/m^2^) were recruited between the months of May and June of the year 2018 and included in the study. Those individuals who presented forced vital capacity (FVC) and forced expiratory values in the first second (FEV_1_) <80% of predicted, those who refused to participate, failed to perform the protocol or missed at least one of the protocols were excluded from the study, as well as those who presented irregularities (files corrupted by system error while recording data) during data analysis.

### Pulmonary function

A Koko DigiDoser (Spire, Longmont, USA) spirometer was used to measure FVC, FEV_1_ and FEV_1_/FVC ratio in healthy subjects according to the ATS/ERS guidelines [14]. The slow vital capacity maneuver (SVC) was used to measure the inspiratory capacity (IC) variable in absolute value to determine the respiration target to be reached during incentive spirometry protocols.

The results were obtained in their absolute and percentage of predicted values according to Pereira et al. (2007) for the Brazilian population [15], and the highest spirometric values among the reproducible curves were considered for analysis.

### Respiratory muscle strength

Respiratory muscle strength was assessed by maximal inspiratory (MIP) and expiratory (MEP) pressures using a digital manometer (NEPEB-LabCare–UFMG, Belo Horizonte, Brazil) with subjects seated on a chair [16]. MIP was measured from residual volume and MEP from total lung capacity. For each evaluation, a maximum of five tests with a one-minute interval between them was considered for data analysis. Data obtained were compared with previous reference values for the Brazilian population [17], and the highest value of each test was considered for analysis.

### Optoelectronic plethysmography (OEP)

For the purpose of analysis, the chest wall was considered totally and divided into three compartments: pulmonary rib cage (RCp), under the action of rib cage muscles; abdominal rib cage (RCa), under the insertional action of diaphragm; and abdomen (AB), under the action of the diaphragm and the expiratory abdominal muscles. The volume variation of the CW and it is compartments as well as the operational volumes [end-inspiratory (EIV) and end-expiratory (EEV) volume variations], to evaluate dynamic lung volumes and hyperinflation, were assessed through optoelectronic plethysmography (BTS Bioengeneering—OEP System—Milan, Italy). The system consisted of eight cameras, previously calibrated at a frequency of 60 Hz, which captured the signal of 89 retroreflective markers placed on specific points of the thorax and abdomen [18]. Volume variations of total CW and its compartments were obtained according to the Gauss Theorem [19].

The subjects were placed in a seated position, with 89 markers anatomically arranged on the chest to capture the variations of CW volumes through the OEP. The subjects remained seated on a backless bench located equidistant from the four anterior and four posterior cameras, with their spine erect, hips and knees bent at 90°, feet resting flatly on the ground, arms supported by abduction at 45°, externally rotated to 90° and elbows flexed at 45°, fists in neutral position and closed hands holding the support bars.

CW tidal volume (V_T,CW_) and its compartments (V_T,RCp_, V_T,RCa_, V_T,AB_) were considered for data analysis. In addition, breathing frequency, minute volume and respiratory times (inspiratory time–Ti, expiratory time–Te and total time of the respiratory cycle- Ttot) were calculated.

### Thoracoabdominal asynchrony

Asynchrony quantification between the CW compartments was calculated by construction of Lissajou figures, previously described in the literature [20, 21]. The phase angle (θ) was defined as the degree of opening of the Lissajou figure when the volumetric signals of the two compartments are plotted against each other, and was defined by the following formula: θ = sin^-1^(m/s), where "*m*" is the distance delimited by the intercepts of the dynamic loop on a line parallel to the X-axis (V_T,AB_) at 50% of the tidal volume of the signal on the Y-axis (V_T,RCp_), and “*s”* represents the tidal volume of the signal on the X-axis. By convention, a θ of zero can be interpreted as perfect synchrony between the evaluated compartments, whereas the value of 180° represents total asynchrony [21, 22].

In addition, the fraction of time in which the RCp, RCa and AB compartment move in opposite directions during inspiration and expiration were calculated and defined as inspiratory (IP) and expiratory paradox time (EP), respectively.

### Study design

The evaluations were distributed into three moments (1, 2 and 3) which were separated by a minimum interval of 48h difference to ensure that the effect of one technique did not overlap into the other (“wash-out effect”). The volume-oriented incentive spirometer (Voldyne 5000, Sherwood Medical, St. Louis/USA) and a spring load with positive expiratory pressure valve with a manually adjustable load with pressure between 5 and 20 cmH_2_O (Mercury Medical, Florida/USA) were used during evaluations. The devices were used separately for evaluation during IS-v and PEP, but were also combined with the aid of a T-tube connection with a unidirectional valve; a combination which in this study was called incentive spirometer volume and pressure oriented (IS-vp).

**Fig 1:**
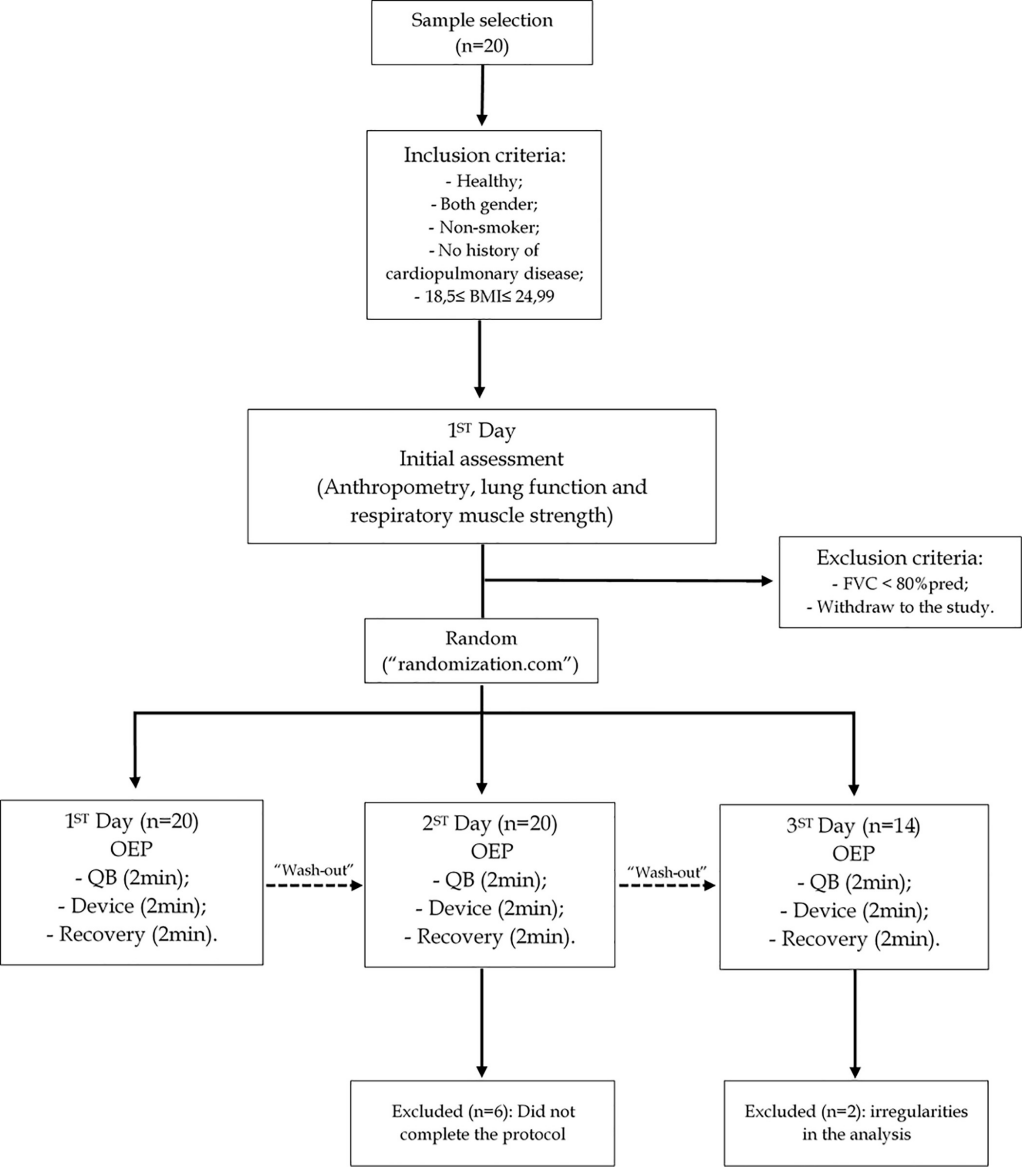
Method flowchart. BMI: Body mass index; FVC: Forced vital capacity; OEP: Optoeletronic plethysmography; QB: Quiet breathing.

At moment 1, the subjects were evaluated for anthropometry, vital signs (blood pressure—BP, heart rate—HR, peripheral oxygen saturation—SpO_2_), respiratory muscle strength (MIP and MEP) and pulmonary function (spirometry). Then the performance order of the pulmonary reexpansion interventions was randomized and the variation of CW volumes were evaluated through OEP, with the first device being performed at this moment occurring in three consecutive stages, on sitting position: 2 minutes of quiet breathing (QB), 2 minutes of randomized pulmonary reexpansion device (intervention) and 2 minutes of recovery. The same was reproduced for moments 2 and 3, using the other randomized devices (Fig 1).

The breathing target in IS-v was defined as 80% of IC, analyzed from the SVC maneuver during spirometry. Breathing with the PEP device was performed without deep inspiratory stimulus and with a load of 10 cmH_2_O, through a mouthpiece, for all subjects based on a previous study [23], only with the instruction of releasing the air overcoming the resistance imposed to the expiratory phase. During the IS-v and IS-vp, the individuals were instructed to perform maximum inspiration through a mouthpiece until reaching 80% of IC as signaled on the spirometer followed by 3-s post inspiratory pause, and again to release the air during the expiratory phase in a free pattern (when performing the IS-v) or against expiratory resistance (when performing the IS-vp protocol). The subjects were not familiar with the devices, but were patiently explained the procedures prior to data acquisition.

### Statistical analyses

Data are shown in mean ± SD or median [interquartile range 25–75%] according to distribution. Data distribution was analyzed using Shapiro-Wilk test. Differences between groups were analyzed using the One-way ANOVA, Two-way ANOVA or Kruskal-Wallis test. In the event of statistically significant differences, Bonferroni or Dunn *post-hoc* tests were applied to identify the difference between groups. Effect sizes were calculated using G*Power software (G*Power 3.1.9.2, Kiel, Germany). Cohen’s f was also expressed (<0.20) as moderate (0.25 and 0.40) or large (>0.40) [24]. Inferential analyses were performed using GraphPadPrism 6.0 (La Jolla, Ca, USA). A significance level of 5% (p <0.05) was adopted for all statistical analyses.

## Results

Twenty (20) healthy individuals were invited to participate in the present study with the objective to evaluate and compare the kinematics of the CW and its compartments in using three different devices for pulmonary reexpansion: IS-v, PEP and IS-vp. Six subjects only attended the first day and therefore did not complete the protocol and two were excluded due to irregularities in the analysis file. Thus, the final sample consisted of 12 individuals, being 6 males and 6 females. Considering the mean of each group and the difference between standard deviation of V_T,CW_ variable while using the devices, a Cohen’s d = 0.79 (considered large) was found and showed a power (1-β) = 0.98 for this study.

Regarding anthropometric characteristics, lung function and respiratory muscle strength, all individuals presented values within the normality patterns (S1 Table), as expected. The mean age was 24.75 ± 2.30 years and subjects had a mean BMI of 22.86 ± 1.37 kg/m^2^, being classified as eutrophic. Regarding lung function the subjects had a mean FVC as a percentage of predicted of 96.16 ± 9.29%, FEV_1_ as a percentage of predicted of 92.99 ± 6.68% and FEV_1_/FVC of 100.98 ± 6.80%. Finally, in relation to the predicted percentage, the individuals had a mean maximal inspiratory pressure of 95.85 ± 16.94% and maximum expiratory pressure of 98.99 ± 15.14%.

### Chest wall volumes and breathing pattern

As shown in Table 1, during pulmonary reexpansion interventions it was possible to observe that there was a significant increase in V_T,CW_ [IS-v: Cohen’s *f* = 1.42; PEP: Cohen’s *f* = 1.17; IS-vp: Cohen’s *f* = 3.17], V_T,RCp_ [IS-v: Cohen’s *f* = 1.49; PEP: Cohen’s *f* = 1.48; IS-vp: Cohen’s *f* = 1.64] and V_T,RCa_ [IS-v: Cohen’s *f* = 0.78; PEP: Cohen’s *f* = 0.88; IS-vp: Cohen’s *f* = 1.27] when compared to quiet breathing (p<0.05). In addition, an increase in the V_T,AB_ (p<0.05, Cohen’s *f* = 1.52) was only found during IS-vp.

**Table 1:** Breathing pattern of healthy individuals at rest, during pulmonary reexpansion interventions and recovery in quiet breathing.

Variables	IS-v			PEP			IS-vp		
	QB	Intervention	Recovery	QB	Intervention	Recovery	QB	Intervention	Recovery
**V**_**T**,**CW (L)**_	0.62 ± 0.12	1.99 ± 0.82*^#^	0.55 ± 0.10^¥^	0.57 ± 0.14	1.66 ± 0.72*^#^	0.65 ± 0.20^¥^	0.55 ± 0.16	2.90 ± 0.45*	0.85 ± 0.36^¥^
**V**_**T**,**RCp (L)**_	0.24 ± 0.07	1.03 ± 0.44*	0.24 ± 0.06^¥^	0.26 ± 0.09	0.83 ± 0.27*^#^	0.32 ± 0.12^¥^	0.22 ± 0.08	1.23 ± 0.41*	0.39 ± 0.25^¥^
**V**_**T**,**RCa (L)**_	0.13 ± 0.04	0.41 ± 0.31*^#^	0.11 ± 0.05^¥^	0.09 ± 0.03	0.33 ± 0.21*^#^	0.11 ± 0.06	0.11 ± 0.06	0.65 ± 0.33*	0.13 ± 0.10^¥^
**V**_**T**,**AB (L)**_	0.25 ± 0.12	0.54 ± 0.34^#^	0.20 ± 0.09^¥^	0.22 ± 0.11	0.47 ± 0.39^#^	0.22 ± 0.12	0.23 ± 0.11	1.01 ± 0.37*	0.33 ± 0.19^¥^
**Ti**_**(s)**_	2.55 ± 0.72	3.53 ± 1.60	2.69 ± 1.12	2.40 ± 0.74	3.20 ± 1.85	2.56 ± 0.70	2.81 ± 0.70	3.58 ± 1.35	2.91 ± 0.68
**Te**_**(s)**_	3.49 ± 1.02	6.88 ± 2.03*	3.65 ± 1.18^¥^	3.15 ± 0.88	4.67 ± 3.61	2.95 ± 0.76	3.69 ± 1.13	6.35 ± 2.85*	3.87 ± 1.98
**Ttot (s)**	6.04 ± 1.53	10.41 ± 1.85*	6.33 ± 2.15^¥^	5.55 ± 1.58	7.87 ± 4.13	5.50 ± 1.34	6.50 ± 1.69	9.93 ± 3.42*	6.78 ± 2.46
**f**_**(bpm-1)**_	10.75 ± 3.07	6.10 ± 1.29*	10.85 ± 3.73^¥^	11.95 ± 3.61	9.45 ± 4.06	11.82 ± 3.30	10.07 ± 3.30	7.42 ± 3.83	9.76 ± 2.83
**VE**_**(L/min-1)**_	8.19 ± 2.25	12 ± 5.35	6.35 ± 3.67	8.71 ± 2.41	12.18 ± 5.81	9.14 ± 3.98	7.08 ± 2.49	14.90 ± 8.65*	6.19 ± 4.54

Data presented as mean ± standard deviation. IS-v: Incentive spirometer volume oriented; PEP: Positive expiratory pressure; IS-vp: Incentive spirometer volume and pressure oriented; QB: quiet breathing; V_T,CW_: Chest wall tidal volume; V_T,RCp_: Pulmonary ribcage tidal volume; V_T,RCa_: Abdominal ribcage tidal volume; V_T,AB_: Abdominal tidal volume; Ti: Inspiratory time; Te: Expiratory time; Ttot: Total time of the respiratory cycle; *f*: breathing frequency; VE: Minute volume; L: Liters; min: minutes; s: seconds; Bpm: Breaths per minute; two-way ANOVA (parametric data distribution)

* <0.05 intervention *versus* QB

^#^ <0.05 IS-v or PEP *versus* IS-vp

^¥^ <0.05 recovery *versus* intervention.

Regarding volume variation displaced during the use of the devices, the IS-vp was able to promote a greater increase (p<0.05) in volume of all compartments when compared to IS-v (V_T,CW_: Cohen’s *f* = 0.81; V_T,RCp_: Cohen’s *f* = 0.53; V_T,RCa_: Cohen’s *f* = 0.50; V_T,AB_: Cohen’s *f* = 0.76). When compared to PEP, an increase in V_T,CW_ (Cohen’s *f* = 0.80) was observed due to an increase in V_T,RCp_ (Cohen’s *f* = 0.53) and V_T,RCa_ (Cohen’s *f* = 0.50) (Table 1).

Te and Ttot were significantly higher (p<0.05) while using IS-v (Te: Cohen’s *f* = 1.10; Ttot: Cohen’s *f* = 1.12) and IS-vp (Te: Cohen’s *f* = 0.60; Ttot: Cohen’s *f* = 0.62). A reduction (p<0.05) of both times in the recovery moment was also observed for IS-v when compared to the other devices (Table 1). Breathing frequency was lower during IS-v when compared to QB and higher when compared to the recovery moment (p<0.05, Cohen’s f = 0.79). Finally, a significant increase in VE was observed only during IS-vp in comparison to QB (p<0.05, Cohen’s *f* = 0.70), as shown in Table 1.

### Thoracoabdominal asynchrony

A significantly lower θ for RCp *versus* AB (p<0.05, Cohen’s *f* = 0.72) and RCp *versus* RCa (p<0.05, Cohen’s *f* = 0.38) were found while using IS-vp when compared to IS-v (Table 2). IP_RCa_ was significantly higher in the recovery moment when compared to using IS-v (p<0.05, Cohen’s *f* = 0.62) and IS-vp (p<0.05, Cohen’s *f* = 0.44) devices (Table 2). Regarding EP, a higher value was found when analyzing RCa during recovery in comparison to using IS-v (p<0.05, Cohen’s *f* = 0.20) (Table 2).

**Table 2:** Chest wall asynchrony at rest, during pulmonary reexpansion interventions and recovery in quiet breathing.

Variables	IS-v			PEP			IS-vp		
	QB	Intervention	Recovery	QB	Intervention	Recovery	QB	Intervention	Recovery
**θ**_**RCpAB (°)**_	8.68 [5.67–20.08]	25.13 [20.41–35.95]	18.76 [4.96–26.78]	6.04 [2.23–17.11]	13.53 [2.20–25.11]	18.48 [4.99–13.58]	10.95 [3.89–22.75]	5.25 [3.17–9.51]*	5.56 [2.01–8.76]
**θ**_**RCpRCa (°)**_	8.76 [3.54–18.93]	10.40 [4.74–21.52]	13.78 [4.06–23.63]	6.31 [1.15–9.78]	7.99 [3.44–18.19]	6.30 [1.87–22.03]	4.74 [2.36–10.13]	4.27 [1.50–7.14]*	3.53 [2.12–5.81]
**θ**_**RCaAB (°)**_	4.19 [2.07–14.87]	10.01 [4.50–22.61]	17.94 [5.20–23.46]	9.34 [3.65–13.23]	10.34 [4.24–15.74]	7.35 [1.91–32.55]	8.79 [1.40–15.04]	12.01 [5.18–21.47]	5.99 [1.54–10.83]
**IP**_**RCp (%)**_	14.17 [12.32–22.02]	7.66 [5.83–11.10]	19.60 [10.42–25.87]	16.38 [8.94–24.14]	13.36 [6.84–18.51]	17.31 [8.32–28.28]	17.03 [9.71–22.25]	7.23 [5.07–12.42]	14.04 [11.59–22.95]
**IP**_**RCa (%)**_	17.44 [12.72–23.72]	7.68 [4.60–15.13]	22.33 [19.1–29.86]^#^	19.55 [13.29–30.03]	13.81 [7.36–22.77]	20.87 [11.47–39.58]	14.55 [9.58–28.73]	8.85 [5.80–11.86]	22.25 [10.8–26.33]^#^
**IP**_**AB (%)**_	17.03 [11.56–18.71]	12.80 [7.10–21.63]	14.43 [9.88–31.97]	16.89 [12–20.88]	18.19 [6.71–25.87]	16.34 [9.25–28.58]	14.95 [11.10–19.48]	17.21 [9.39–21.54]	17.26 [12.34–23.71]
**EP**_**RCp (%)**_	17.78 [12.36–27.29]	11.44 [7.35–12.45]	19.19 [11.77–24.90]	19.43 [10.88–26.13]	12.50 [7.73–19.53]	17.87 [8.57–25.01]	18.28 [14.93–25.93]	12.35 [7.71–15.26]	14.11 [9.53–27.43]
**EP**_**RCa (%)**_	19.65 [16.39–25.27]	14.32 [9.98–19.84]	25.27 [23.6–31.75]^#^	26.46 [14.50–33.40]	19.36 [10.36–25]	19.57 [13.16–33.20]	14.94 [9.80–28.34]	16.34 [15.06–23.82]	19.32 [9.38–31.86]
**EP**_**AB (%)**_	16.46 [8.81–20.75]	7.48 [4.94–16.87]	19.80 [9.50–30.83]	19.99 [13.12–26.47]	17.12 [8.04–24.87]	17.95 [8.99–27.31]	15.93 [11.97–19.64]	16.05 [6.13–22.70]	12.39 [6.66–25.13]

Data presented as median and interquartile range between 25–75%; IS-v: Incentive spirometer volume oriented; PEP: Positive expiratory pressure; IS-vp: Incentive spirometer volume and pressure oriented; QB: quiet breathing; θ_RCpAB_: Phase angle between pulmonary rib cage and abdomen compartments; θ_RCpRCa_: Phase angle between pulmonary rib cage and abdominal rib cage compartments; θ_RCaAB_: Phase angle between abdominal rib cage and abdomen compartments; IP_RCp_: Inspiratory paradox time in percentage of pulmonary rib cage compartment; IP_RCa_: Inspiratory paradox time in percentage of the abdominal rib cage compartment; IP_AB_: Inspiratory paradox time in percentage of the abdomen compartment; EP_RCp_: Expiratory paradox time in percentage of pulmonary rib cage compartment; EP_RCa_: Expiratory paradox time in percentage of the abdominal rib cage compartment; EP_AB_: Expiratory paradox time in percentage of the abdomen compartment; Kruskal-Wallis test (non-parametric data distribution)

* <0.05 *versus* IS-v during the intervention

^#^ <0.05 *versus* intervention.

### Operational chest wall and compartmental volumes

When analyzing IS-v, significant differences between EIV versus EEV in CW and its compartments were observed during QB (p<0.05) while pulmonary reexpansion intervention (p<0.05) and only during recovery for CW, RCp and AB (p<0.05). EEVcw, EEV_RCa_ and EEV_AB_ decreased during IS-v when compared to QB (p<0.05), while a significant increase in EIV_CW_, EIV_RCp_ and EIV_RCa_ (p<0.05) and decreases in EIV_AB_ (p<0.05) were observed. During the recovery moment, significant decreases in EIV_CW_ and EIV_RCp_ (p<0.05) were observed when compared to the IS-v device, as well as significant increases in EEV_CW_, EEV_RCa_ and EEV_AB_ (p<0.05). It was possible to observe that EIV_RCa_ and EEV_RCa_ were significantly higher when comparing recovery to QB (p<0.05).

The same pattern was found regarding PEP when analyzing EIV and EEV in three moments (p<0.05) for all compartments. EEV_RCp_ presented a significant difference between devices versus QB (p<0.05) and recovery versus QB (p<0.05), while EIV_CW_ and EIV_RCp_ were significantly different (p<0.05) for the same comparison.

**Fig 2:**
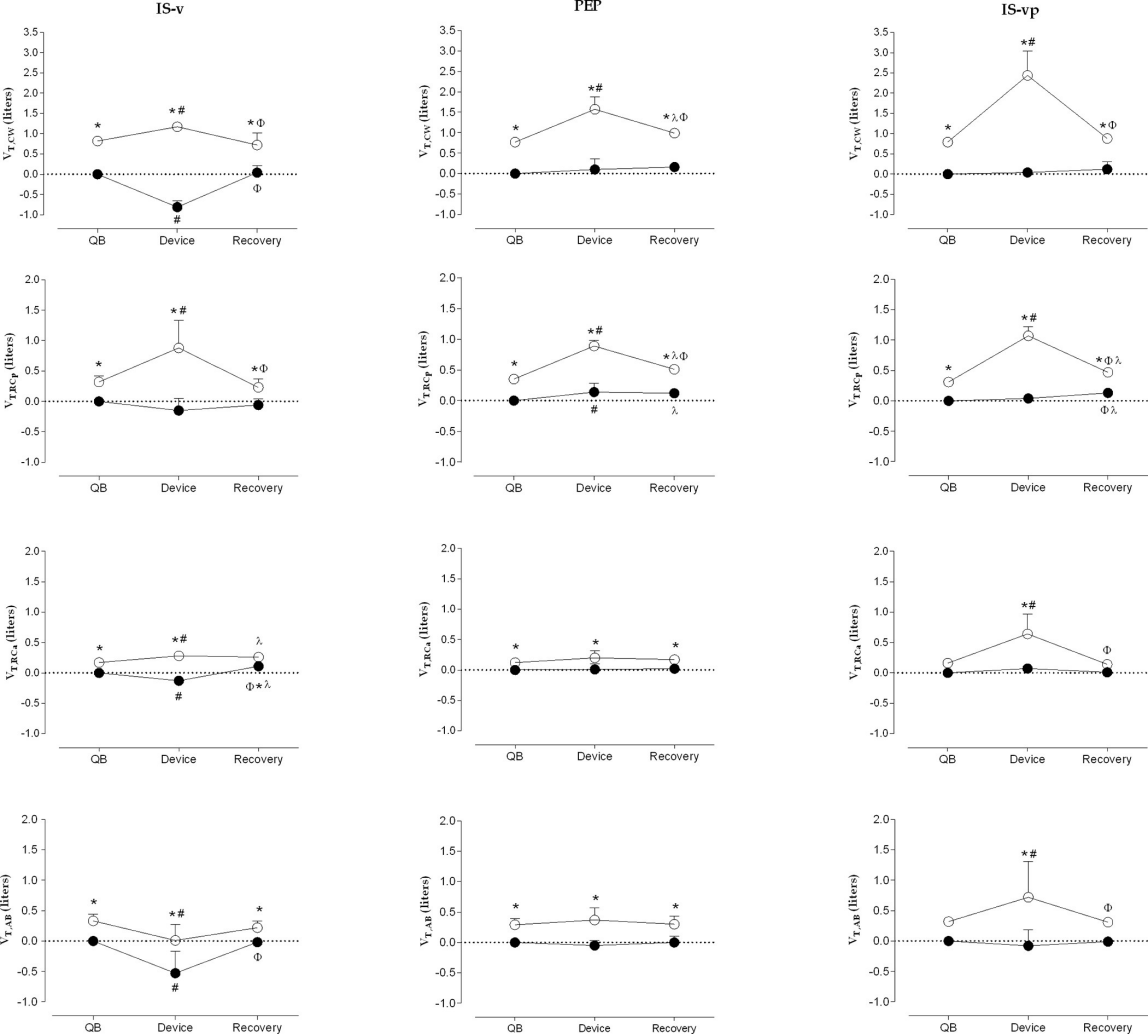
Operating total and compartmental chest wall volumes at rest, during pulmonary reexpansion interventions and recovery in quiet breathing. Data presented as mean ± standard deviation. IS-v: Incentive spirometer volume-oriented; PEP: Positive expiratory pressure; IS-vp: Incentive spirometer volume and pressure oriented. QB: quiet breathing; V_T,CW_: Chest wall tidal volume; V_T,RCp_: Pulmonary ribcage tidal volume; V_T,RCa_: Abdominal ribcage tidal volume; V_T,AB_: Abdominal tidal volume. White dot: End-inspiratory volume (EIV); Black dot: End-expiratory volume (EEV). two-way ANOVA (parametric data distribution); * <0.05 EIV *versus* EEV; # <0.05 device *versus* QB; Φ: recovery *versus* intervention; λ: recovery *versus* QB.

Significant statistical differences were found between EEV and EIV using IS-vp in the three moments for only CW and RCp (p<0.05), and while using the device for RCa (p<0.05) and AB (p<0.05). Regarding EIV during pulmonary reexpansion intervention in comparison to QB, higher values were observed in CW (p<0.05), RCp (p<0.05), RCa (p<0.05) and AB (p<0.05), and all these volumes were lower when comparing recovery to pulmonary reexpansion intervention moment (p<0.05), and higher regarding EEV only for RCp (p<0.05). Additionally, pulmonary rib cage EEV (p<0.05) and EIV (p<0.05) presented higher values when comparing recovery to QB, as shown in Fig 2.

## Discussion

The main results of the present study were: 1) the IS-vp device was able to generate larger volume variations when compared to using it separately from the other two; 2) During pulmonary reexpansion moments, individuals presented lower thoracoabdominal asynchrony with IS-vp when compared to IS-v; 3) A greater inspiratory paradox time (IP) was found for the abdominal rib cage compartment after using the IS-v and IS-vp and a greater expiratory paradox time (EP) only after using the IS-v also in the abdominal rib cage; 4) EIV was higher when using the three devices for all compartments, except in abdominal compartment using IS-v and pulmonary rib cage EIV and EEV were higher in recovery compared to quiet breathing for PEP and IS-vp, and abdominal rib cage EIV and EEV for IS-v.

To the best our knowledge, this is the first study to compare three different pulmonary reexpansion interventions through OEP. Although incentive spirometer is subdivided into two groups, flow (IS-f) and volume-oriented (IS-v), we decided to only use IS-v because the literature already demonstrates that this device allows the subject to gain more lung volume at the expense of less respiratory effort [25, 26]. Barbalho-Moulin et al. [27] evaluated 28 women undergoing bariatric surgery in order to compare the effects of incentive spirometer flow-oriented (IS-f) and PEP (10 cmH_2_O) on pulmonary function. The authors found that IS-f was more effective in maintaining tidal volume and greater thoracoabdominal motion when compared to PEP group. Although it was also found that devices involving incentive spirometer further increased the tidal volume in the present study, the methodologies differ by type of protocol, since Barbalho-Moulin et al. [27] used IS-f instead of IS-v, and a less accurate methodology for evaluating the volume variation and thoracoabdominal mobility, such as spirometry and thoracoabdominal cirtometry.

Regarding the use of PEP, in the present study the value of 10 cmH_2_O was based on a previous study [23] of patients with Parkinson's disease and restrictive ventilatory pattern, in which no differences were observed between 3 different PEP intensities (10, 15 and 20 cmH_2_O) in increasing the CW volume variation. The authors hypothesized that the 10 cmH_2_O intensity would be more comfortable and safer for the subjects. A recent study [28] evaluated healthy older subjects to assess acute effects of IS-v (self-paced) and PEP (10 to 20 cmH_2_O) using electrical impedance tomography. The authors concluded that both devices promote lung ventilation and recruitment in individuals after surgery, with greater values for end-inspiratory and end-inspiratory volumes while using the devices. However, the authors do not describe in methodology whether all subjects used the same pressure load during PEP, different from our subjects who were conditioned to PEP of 10 cmH_2_O. As in the present study, a period of at least 48 hours was observed to ensure the wash-out effect between the different devices, whereas Reychler et al. [28] only implemented a 5-minute rest period between the maneuvers, which may justify the increased volume of one device over the other.

Based on the studies presented so far and on the findings of the present study, it is possible to observe that the incentive spirometers have a better mobilization of the lung volumes when compared to the PEP applied alone, and that this can be influenced by the pressure level offered. It can also be inferred that the incentive spirometers seem to have a better result because of the inspiratory stimulus that is given for the series realization.

Another group of authors [11, 29] evaluated the effects of incentive spirometry associated with positive expiratory pressure, as in the present study. The authors found a greater recovery of strength and lung function, shorter hospitalization time and lower incidence of pulmonary complications in the group that used the combined devices when compared to the group that only received instructions for ventilatory patterns and coughing, as well as improvement in the sensation of dyspnea and functional capacity. It is important to report that the authors did not assess volume variations of chest wall by OEP as was evaluated in the present study, which makes impossible a more reliable comparison between the findings. Individuals who undergo surgical procedures involving anesthesia may have postoperative pulmonary complications (PPCs) including atelectasis, pleural effusion and pneumonia. In addition, surgical manipulation and the presence of postoperative pain also influence altered ventilatory pattern and the appearance of such complications [30, 31]. Chest physiotherapy aims to reduce these complications, mainly through respiratory exercises, incentive spirometer and administration of positive pressures in the airways. In addition, we can not rule out the use of early mobilization protocols and position changes, which act as sources of sensory-motor stimulation, but also prevent pulmonary complications secondary to immobilization [10].

Regarding thoracoabdominal asynchrony, in a recent study the authors compared the use of IS-f and IS-v in older adults (70.6 ± 2.3 years) and young individuals (25.9 ± 4.7 years) through OEP [32]. An increase in chest wall volume variation and all compartments was observed during IS-v when compared to quiet breathing (p<0.05), whereas no significant increase was observed in the abdominal compartment in the present study. The increase in volume was accompanied by a greater synchrony between the pulmonary rib cage and abdominal compartments when the IS-v was compared to the IS-f. In the present study, the subjects presented greater asynchrony exactly during the IS-v when compared to IS-vp [25.13 (20.41–35.95) *versus* 5.25 (3.17–9.51), p<0.05, respectively]. IP_RCa(%)_ and EP_RCa(%)_ were greater in our study after using IS-v, which may explain why the abdominal compartment did not show a significant increase for IS-v.

In individuals with chest wall restriction, low compliance increases the minute volume at the expense of a higher breathing frequency. It is known that the aim of incentive spirometry is to increase transpulmonary pressure through slow deep maximal inspiration, increasing tidal volume and decreasing breathing frequency. In our study subjects had a greater VE (p<0.05) only during IS-vp performance when compared to quiet breathing due to a larger tidal volume associated with a low breathing frequency, which led to achieving an expansibility gain in the inspiratory phase. Additionally, an association of positive expiratory pressure seems to promote collateral ventilation and prevent airway collapse during expiratory phase, providing a greater increase than using devices separately.

A recent study with post-stroke subjects evaluated the influence of IS-v on chest wall volumes and mobility [7]. The authors found that IS-v promotes an increase in volume variation in the chest wall when compared to quiet breathing, especially due to an increase in V_T,RCp_. Lower asymmetry between the paretic and healthy hemithorax using IS-v was also found, but the asynchrony between compartments was not evaluated as in the present study. When evaluating the same population as Lima et al. [7], Cabral et al. [33] observed that the group of post-stroke subjects presented a low increase in the V_T,CW_ when compared to quiet breathing during PEP performance. In this case, the increase was considered due to a greater contribution of the abdominal compartment. The authors also found an increase in operational volumes (EEV and EIV) in the post-stroke group, similar to what was found in the present study.

In a study involving patients with cystic fibrosis and healthy children during the use of two PEP intensities (10 and 20 cmH_2_O) for evaluating chest wall volume variation through OEP [4], the authors found similar results to the present study for EIV, which not only presented a significant increase during the PEP, but also while using the other two devices–except for the abdominal compartment during the IS-v–and also for the EEV during PEP. However, a significant reduction of EEV was observed in the chest wall, abdominal rib cage and abdominal compartment using IS-v. The IS-v device was able to mobilize greater volume variation in the pulmonary rib cage compartment, even without significant statistical change when compared to PEP alone, which may have influenced these results.

It is important to highlight, despite the results found, from the clinical point of view, that the pulmonary expansion devices used in the present study have methodological differences regarding the instruction for the use, which may have influenced the results. The increase in pulmonary expansion, therefore, also occurs physiologically differently. Incentive spirometer volume-oriented is based on increased lung expansion through reduction of pleural pressure, using visual stimulus to achieve pre-determined volumes for each subject (80% of IC), followed by a post-inspiratory pause [7]. On the other hand, the PEP valve promotes lung expansion due to increase in alveolar pressure during expiratory phase. The subjects are instructed to overcome the expiratory load, which keeps the airways ventilated for a longer period at the expense of a positive pressure [34]. Therefore, although the results of the present study show differences in chest wall kinematics, they reflect the specific instructions that are directed during the use of each device for pulmonary reexpansion.

The literature still lacks studies comparing the efficacy of different ways of promoting pulmonary reexpansion through accurate evaluation methods, as is the case of OEP.

It is important to emphasize that this is the first study that has included the combined use of two devices and compared the acute effects through optoelectronic plethysmography in healthy subjects. Perhaps the results were underestimated while performing PEP because the offered pressure was low for the evaluated subjects. We suggest that further research be done comparing the same protocol with other pressure loads in healthy subjects and that the effects in patients with restrictive lung diseases who benefit greatly from pulmonary reexpansion techniques should be evaluated.

## Conclusion

Based on the findings of the present study, it can be concluded that the IS-vp is able to generate larger volumes in the chest wall and its compartments, thus promoting lower thoracoabdominal asynchrony in healthy subjects. The findings are of great clinical importance, since IS-vp may be indicated for patients with restrictive respiratory pattern and could require an increase in pulmonary volumes, and consequently better performance of daily life activities and quality of life if used in the long term.

## Supporting information

S1 Table Anthropometric, lung function and respiratory muscle strength data for all individuals. BMI: Body mass index; FVC: Forced vital capacity; FEV_1_: Forced expiratory volume in the first second; MIP: Maximal inspiratory pressure; MEP: Maximal expiratory pressure; M: Male; F: Female; m: Meters; kg: Kilograms; %pred: Predicted percentage.
(PDF)
