# Supplementary material for: Acute effects of three pulmonary reexpansion modalities on thoracoabdominal motion of healthy subjects: Randomized crossover study
Source: PLoS One. 2019 Mar 19;14(3):e0213773. doi: 10.1371/journal.pone.0213773 (PMC6424428; doi:10.1371/journal.pone.0213773)
Supplement: S1 Table — BMI: Body mass index; FVC: Forced vital capacity; FEV1: Forced expiratory volume in the first second; MIP: Maximal inspiratory pressure; MEP: Maximal expiratory pressure; M: Male; F: Female; m: Meters; kg: Kilograms; %pred: Predicted percentage. (PDF) [file pone.0213773.s001.pdf]

Table 1. Anthropometric, lung function and respiratory muscle strength data for all individuals.

Subject	Gender	Age (years)	Height (m)	Weight (kg)	BMI (kg/m2)	FVC (%pred)	FEV_1 (%pred)	FEV_1/FVC (%)	MIP (%pred)	MEP (%pred)
**#1**	M	24	1.71	70	23.94	99.80	85.58	92.55	71.27	102.15
**#2**	M	27	1.72	71	24.00	91.62	90.28	96.47	99.48	78.78
**#3**	M	22	1.75	71	23.18	102.80	95.12	113.25	75.53	74.58
**#4**	M	24	1.67	69	24.74	92.74	91.47	98.78	79.35	78.15
**#5**	M	28	1.81	73	22.28	95.71	98.92	106.02	101.58	105.87
**#6**	M	29	1.79	79	24.66	88.70	83.71	102.38	102.20	105.07
**#7**	F	23	1.65	58	21.30	110.78	102.68	95.24	90.79	105.36
**#8**	F	22	1.64	55	20.45	109.69	103.31	96.39	131.50	117.44
**#9**	F	25	1.62	61	23.24	96.47	96.11	101.19	96.79	109.61
**#10**	F	24	1.69	64	22.41	100.96	95.10	95.24	114.56	111.92
**#11**	F	26	1.62	56	21.34	83.42	84.74	101.18	101.37	112.29
**#12**	F	23	1.65	62	22.77	81.28	88.82	113.10	85.75	86.64
**Mean ± SD**		24.75 ± 2.30	1.69 ± 0.06	65.75 ± 7.52	22.86 ± 1.37	96.16 ± 9.29	92.99 ± 6.68	100.98 ± 6.80	95.85 ± 16.94	98.99 ± 15.14

BMI: Body mass index; FVC: Forced vital capacity; FEV_1: Forced expiratory volume in the first second; MIP: Maximal inspiratory pressure; MEP: Maximal expiratory pressure; M: Male; F: Female; m: Meters; kg: Kilograms; %pred: Predicted percentage.
